# Domain-specific hearing-in-noise performance is associated with absolute pitch proficiency

**DOI:** 10.1038/s41598-022-20869-2

**Published:** 2022-09-29

**Authors:** I-Hui Hsieh, Hung-Chen Tseng, Jia-Wei Liu

**Affiliations:** 1 Institute of Cognitive Neuroscience, National Central University, No. 300, Zhongda Rd., Zhongli District, Taoyuan City, 320317 Taiwan; 2 Cognitive Intelligence and Precision Healthcare Center, National Central University, No. 300, Zhongda Rd., Zhongli District, Taoyuan City, 320317 Taiwan

**Keywords:** Psychology, Human behaviour

## Abstract

Recent evidence suggests that musicians may have an advantage over non-musicians in perceiving speech against noisy backgrounds. Previously, musicians have been compared as a homogenous group, despite demonstrated heterogeneity, which may contribute to discrepancies between studies. Here, we investigated whether “quasi”-absolute pitch (AP) proficiency, viewed as a general trait that varies across a spectrum, accounts for the musician advantage in hearing-in-noise (HIN) performance, irrespective of whether the streams are speech or musical sounds. A cohort of 12 non-musicians and 42 trained musicians stratified into high, medium, or low AP proficiency identified speech or melody targets masked in noise (speech-shaped, multi-talker, and multi-music) under four signal-to-noise ratios (0, − 3, − 6, and − 9 dB). Cognitive abilities associated with HIN benefits, including auditory working memory and use of visuo-spatial cues, were assessed. AP proficiency was verified against pitch adjustment and relative pitch tasks. We found a domain-specific effect on HIN perception: quasi-AP abilities were related to improved perception of melody but not speech targets in noise. The quasi-AP advantage extended to tonal working memory and the use of spatial cues, but only during melodic stream segregation. Overall, the results do not support the putative musician advantage in speech-in-noise perception, but suggest a quasi-AP advantage in perceiving music under noisy environments.

## Introduction

Hearing-in-noise (HIN) perception is an important ability in our daily life. Difficulties in HIN are often associated with aging^1,2^, hearing impairment^3^, and language problems in children^4^, affecting daily communication and sometimes leading to emotional stress. Existing evidence suggests that attention, auditory working memory, or musical experience is associated with an enhanced ability to comprehend speech in a challenging listening environment^5,6^. In particular, musicianship is one of the factors that has been linked to better HIN performance over the years, though some controversies remain^5,7^. The idea of a musician enhancement is attractive because it suggests a possibility that musical training could be used to alleviate problems associated with HIN perception. Additionally, it addresses an important issue underlying the larger question of whether the perceptual and neural changes related to music training can transfer to a non-music domain, such as language^8^.

Several studies have suggested a musician advantage in understanding speech in a noisy environment^5,7^_._ These studies typically compared musicians and non-musicians on speech-in-noise (SIN) performance using materials such as sentences or words embedded in different types of background sounds. A musician advantage has been reported under different types of background noise, such as speech-spectrum noise or multiple talkers^9–14^, and when speech and noise were presented from the same spatial location or in a naturalistic 3D environment^15–18^. In addition to speech materials, Coffey et al.^19^ demonstrated that musicians outperformed non-musicians in identifying a target melody embedded in a noise background of multi-melodies. On the other hand, several studies reported that musicians and non-musicians were equally adept at identifying target sentences when embedded in different types of modulated maskers^20^, separated by fundamental frequency differences from noise streams^21^ or spatially separated with speech maskers^22,23^. In addition, among the studies that have reported differences in HIN perception between musicians and non-musicians, the effects have generally been small^21,24,25^.

One of the reasons for the observed discrepant findings across studies may be the individual differences in perceptual or cognitive abilities associated with musicianship. Specifically, musicians have been documented to differ from non-musicians in several cognitive and perceptual abilities, such as a finer pitch discrimination threshold and enhanced auditory working memory (AWM)^26,27^. In addition, musicians may have certain superior auditory skills or innate musicality that prompt them to pursue professional music training in the first place. Thus, superior HIN performance associated with highly-trained musicians may be partially accounted for by these perceptual differences or preexisting factors^28^. However, almost all previous studies on the musician advantage in HIN perception have compared musicians and non-musicians as two homogenous groups, leaving the individual variabilities in perceptual abilities related to musicianship unexplored. Given the diverse nature of musicianship, it remains to be determined which aspect(s) associated with musicians may critically contribute to their enhanced HIN ability.

In this study, we tested the hypothesis that a comparatively stable trait of musicians, absolute pitch (AP) proficiency, can partially account for the musician advantage in HIN performance. We chose to examine AP ability, defined as the ability to name and produce an isolated musical note without a referent^29^, for the following reasons. First, AP is one of the few defining traits of musicians shown to both have a genetic origin and require a critical period during development^29–32^. Second, some studies have suggested that AP ability is trainable only up to a certain level compared to relative pitch (RP) ability^33^. In addition, there’s recent indication that AP proficiency is associated with an enhanced ability to extract musical streams out of interleaved melodies^6^. However, their tasks were limited to musical materials presented in silence; the proficiency of AP ability in relation to HIN performance with speech or musical materials has not been examined. In recent years, quasi-AP, a term that describes the continuum of varying degrees of “intermediate” AP ability observed among the general population, has gained considerable research interest^34–37^. This notion of quasi-AP is also known as implicit or latent AP and considers the ability as more widely existing, in contrast to the stringent criteria which restrict AP to 1–4% of individuals. Hence, we consider here the idea that precision of HIN perception is linked to the “intermediate” levels of AP proficiency, i.e., quasi-AP abilities.

The goal of the current study was to investigate whether a musician advantage exists in HIN perception in both language and music domains, and to determine the degree to which linguistic and musical HIN performance is modulated by the intermediate levels of AP proficiency. A large cohort (N = 54) of listeners, comprising 42 musicians and 12 non-musicians stratified into high, medium, and low AP proficiency groups completed both the Music-in-Noise Task (MINT)^19^ and the Mandarin Hearing-in-Noise Test (M-HINT)^12^. Given that HIN benefits are associated with auditory working memory^5,7,38^ and the use of visuo-spatial cues^5,19^, we also examined these relationships with respect to quasi-AP proficiency. To control for potential covariates to AP abilities, we assessed pitch adjustment and relative pitch abilities in addition to selecting musicians with comparable musical backgrounds. AP abilities have been previously shown to provide benefits to auditory streaming with interleaved melodies^6^. However, it remains unknown whether AP abilities play a role in HIN perception, which similarly involves the process of stream segregation. If musicians rely on AP abilities for auditory stream segregation, then their advantage should extend to HIN perception, irrespective of whether the streams are speech or musical sounds. In addition, AP abilities might provide benefits to cognitive abilities (i.e., AWM, use of visuo-spatial cues) that play a role in stream segregation. The AWM and visuo-spatial cue subtasks were manipulated to introduce comparable music and linguistic materials to further substantiate the hypothesized domain-general AP effect on HIN perception.

We reported a surprising domain-specific AP effect on HIN performance: better quasi-AP abilities were associated with improved perception of melodies in noise, but not of speech in noise. The quasi-AP advantage extends to the use of spatial cues and is related to enhanced AWM recall for tonal materials, but only during melodic stream segregation. Our results provide evidence against the hypothesis that musical training leads to improved speech-in-noise perception, but suggest a quasi-AP (rather than musicianship per se) advantage for music perception under noisy environments.

## Results

### Participants’ characteristics

**Figure 1:**
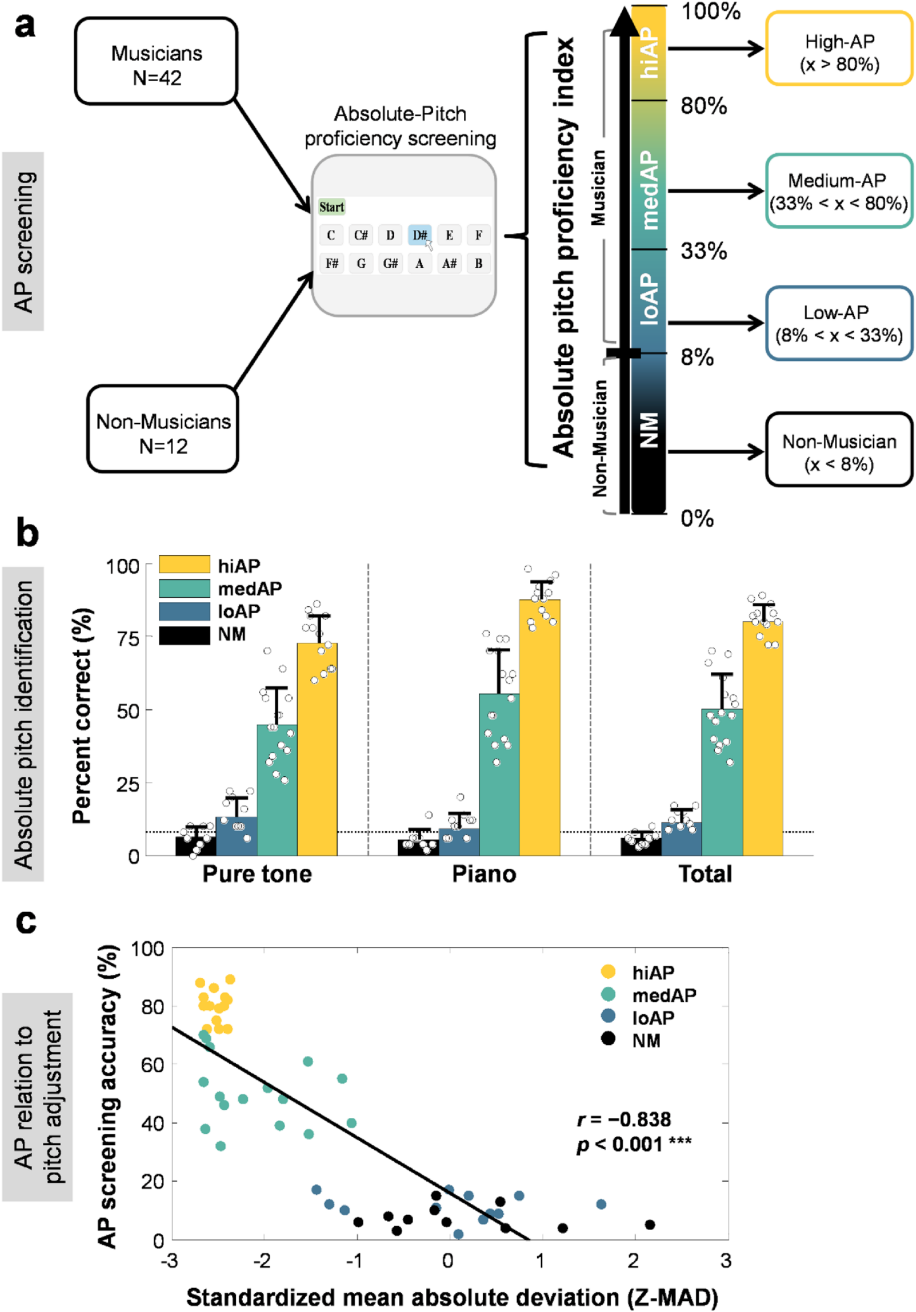
Performance on absolute pitch screening and pitch adjustment for the participants grouped by levels of absolute pitch proficiency. (**a**) Absolute pitch proficiency index. Participants were separated into four groups based on the gradient performance on AP screening tasks. High-AP: 80% or above; med-AP: 33–80%; low-AP: 8–33% and non-musicians: below 8% (chance performance). (**b**) Percent correct performance on absolute pitch identification of pure tone and piano for the high-AP, med-AP, low-AP musicians, and non-musician groups. (**c**) Relationship between pitch adjustment performance (standardized mean absolute deviation) and AP screening score for the four AP proficiency groups. Higher scores on AP screening are associated with smaller mean absolute deviation from target-tone frequency on pitch adjustment task. Z-MAD: z transformed mean absolute deviation in semitones normalized in reference to the non-musician group.

Musicians and non-musicians (NM) were separated into four groups based on AP screening accuracy (i.e., high AP: 80% or above; medium AP: 33–80%; low AP: 8–33%; and non-musicians: below 8% (chance performance); Fig. 1a). These AP proficiency cutoff criteria were in general accordance with previous studies (see Supplementary Table S1)^36,39^. AP screening was performed using a pitch identification task consisting of 50 pure tones and 50 piano notes randomly selected from the equal-tempered scale ranging from C2 to B6 (65.4 to 1975.5 Hz). A pitch adjustment task, which quantifies fine-grain differences in AP proficiency, was performed to validate AP proficiency^6^. A non-musician group was included to facilitate comparison with previous studies on the musician advantage, which typically compared performance differences between musicians and non-musicians. The non-musicians were generally at or below chance performance (i.e., All but three listeners in the non-musician group scored below 8% on the AP screening).

To ensure that the musical backgrounds were comparable between all groups of musicians, we first assessed the onset age and musical training hours across the three levels of AP-proficiency musician groups using a one-way ANOVA. No difference in the musician groups with respect to the age at which formal music training began (*F*(2,39) = 2.415, *p* = 0.053) or total practice hours (*F*(2,39) = 1.028, *p* = 0.367) was revealed. As expected, the four groups differed in terms of AP screening score (*F*(3,50) = 229.218, *p* < 0.001; Fig. 1b) and pitch adjustment accuracy (*F*(3,50) = 48.232, *p* < 0.001; see Supplementary Fig. S1). Mean absolute deviation from target tones (MAD; *z*-transformed) for pitch adjustment correlated significantly with AP screening scores (*r* =  − 0.833, *p* < 0.001; Fig. 1c), validating the AP proficiency level. Post-hoc comparisons with Bonferroni correction across different AP-proficiency groups showed that screening accuracy differed significantly (*p* < 0.001) between all pairs of groups, except for the comparison between the low-AP and the non-musician groups (*p* = 0.167; see Supplementary Table S2). Similarly, pairwise comparisons showed that pitch adjustment accuracy differed significantly between all of the AP proficiency groups, except for the comparisons between the high-AP vs. med-AP group, and between the low-AP vs. the non-musician group (all *ts* >  − 9.105, *ps* < 0.001; see Supplementary Table S3).

We tested whether a group difference exists in AWM ability given previous demonstration of superior AWM performance associated with musicianship^38^. We found no group difference in recall of linguistic materials (*F*(3,50) = 0.517, *p* = 0.673). However, there was a significant main effect of group in AWM ability to recall tonal materials (*F*(3,50) = 19.357, *p* < 0.001). Post-hoc t-tests revealed that recall accuracy on tonal materials differed significantly between the high-AP, med-AP, low-AP groups, and the non-musicians (high-AP > NM, med-AP > NM, and low-AP > NM with all *t*s > 3.551, *ps* < 0.001; see Supplementary Table S4). Given the co-occurrence of AP and RP abilities, we also tested whether the four groups differ in terms of RP abilities using an interval identification task. We found a significant group difference in RP ability (*F*(3,50) = 64.721, *p* < 0.001), with the musician groups overall performing above 80% at identifying RP intervals. Post-hoc comparisons revealed RP ability differences between the high-AP, med-AP, low-AP groups, and the non-musicians (high-AP > NM, med-AP > NM, and low-AP > NM with all *t*s > 6.443, *ps* < 0.001; see Supplementary Table S5). Table 1 presents a summary of general group differences on participants’ characteristics.

**Table 1:** Participants’ characteristics.

	High-AP musicians	Med-AP musicians	Low-AP musicians	Non-musicians
No	14	16	12	12
Age	20.21 ± 1.05	20.38 ± 0.81	22.08 ± 2.19	21.0 ± 0.74
Gender (female/male)	6/8	11/5	4/8	4/8
Handedness (right/left)	13/1	14/2	11/1	12/0
Starting age	5.71 ± 1.90	5.38 ± 1.31	5.75 ± 2.34	N/A
Years of playing	11.29 ± 3.52	9.66 ± 2.68	9.92 ± 3.70	N/A
Total practicing hours	6074 ± 1446.4	4104 ± 1373.4	4981 ± 1264.5	N/A
Primary training style	Classical	Classical	Classical	N/A
Primary instrument	Piano	Piano	Piano	N/A
Screening AP score (**%)**^b^	85.0 ± 5.6	50.1 ± 11.8	11.3 ± 4.3	8.4 ± 3.9
Score range (max = 100**)**	(78–96)	(33–70)	(9–17)	(3–13)
AWM score (word)	0.9 ± 0.07	0.93 ± 0.07	0.92 ± 0.08	0.9 ± 0.09
AWM (tonal)	0.94 ± 0.07	0.95 ± 0.05	0.86 ± 0.08	0.76 ± 0.10
Pitch Adjustment Test (PAT)^c^	0.33 ± 0.09	0.67 ± 0.46	2.40 ± 0.74	2.40 ± 0.82
Relative Pitch task (RP)	1.00 ± 0.00	0.91 ± 0.09	0.83 ± 0.17	0.42 ± 0.20

^a^All scores except screening reported as proportion correct given as Mean ± SD.

^b^Score given in percentage combined from pure tones and piano notes (see “Methods”).

^c^Mean deviation in semitone.

### A domain-specific musician advantage on HIN

**Figure 2:**
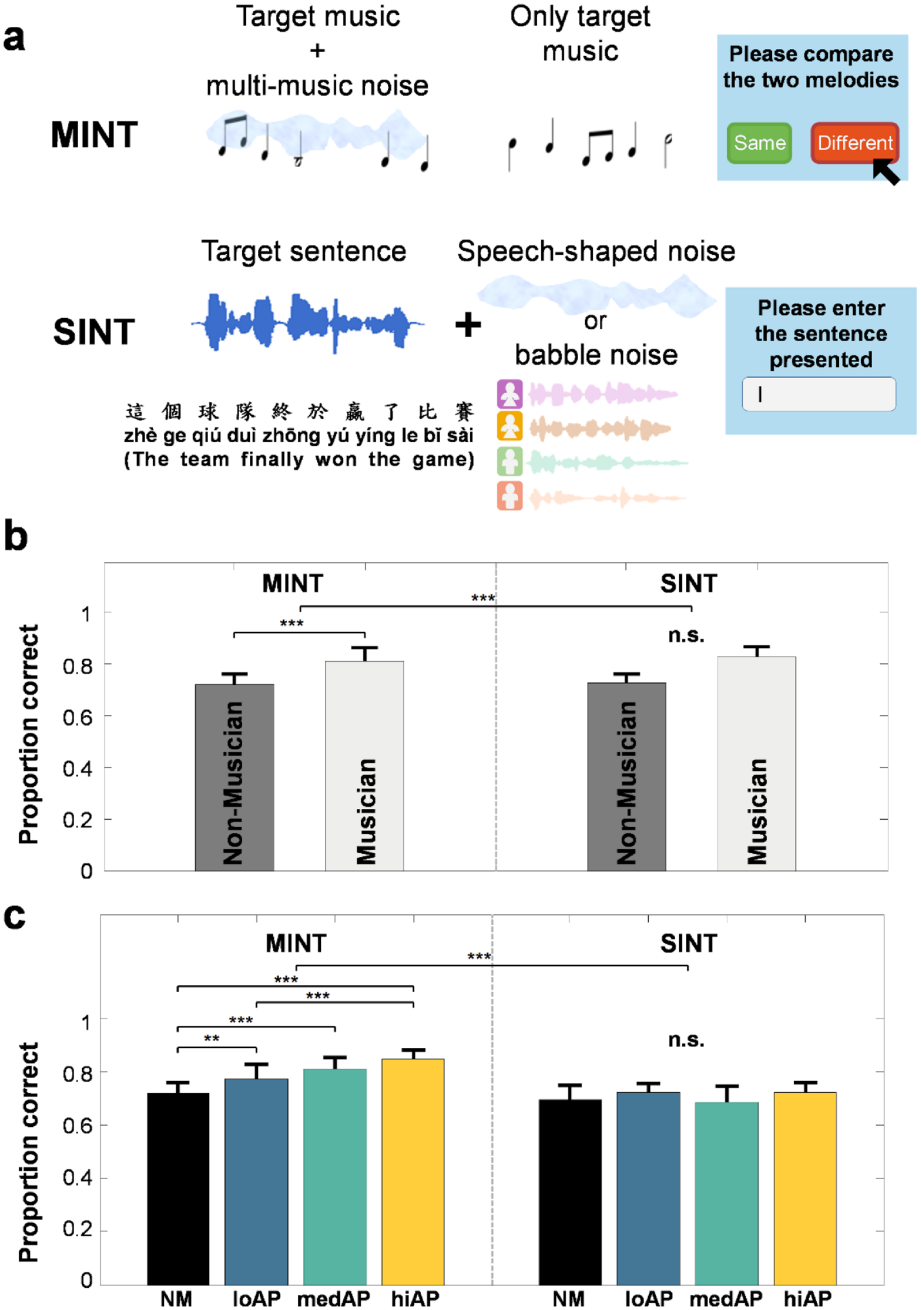
Average performance accuracy on music-in-noise and speech-in-noise tasks as a function of musicianship and quasi-AP proficiency level. (**a**) Schematic diagram of the music-in-noise and speech-in-noise tasks. (**b**) Mean proportion correct on MINT and SINT performance by musician and non-musician groups. (**c**) Mean performance accuracy on MINT and SINT separated into 4 groups based on quasi-AP proficiency level. Error bars represents ± 1 SD. ***p < 0.001, **p < 0.01, *p < 0.05 level of significance; *n.s.* not significant.

We first tested whether a musician advantage exists in HIN that is generalized to both linguistic and musical domains, in an attempt to replicate the previous musician advantage reported in SIN and to assess the hypothesized domain-general HIN advantage. For this analysis, the high-AP, med-AP, and low-AP musicians were first combined into one group–musicians–for comparison with the non-musician group. For linguistic HIN, participants identified 10-word target sentences from the M-HINT embedded in speech-shaped noise at four signal-to-noise ratios (SNR; 0, –3, –6, and –9 dB). Speech-in-noise task (SINT) accuracy was measured as proportion of words correctly reported. For musical HIN, participants judged whether two musical excerpts, one presented in multi-music noise at compatible SNRs followed by the other presented in silence, were identical (Fig. 2a). Music-in-noise task (MINT) accuracy was measured as proportion of melodies correctly identified. Figure 2 shows mean proportion correct for the musician and non-musician groups on the MINT and SINT. A 2 × 2 mixed ANOVA was conducted with the factors musicianship (musician vs. non-musician) and domain (music vs. speech) on HIN accuracy performance. Levene’s test for homogeneity of variance indicated equal variances for the musician and non-musician groups (*F*(1,52) = 0.081, *p* = 0.778). A significant effect of musicianship on HIN performance accuracy was found (*F*(1,52) = 38.857, *p* < 0.001; Fig. 2b). There was a significant main effect of domain on HIN performance, with higher accuracy on the music domain than on the speech domain (*F*(1,52) = 21.317, *p* < 0.001; Fig. 2b). The analysis showed a significant interaction between musicianship and task domain on HIN performance (*F*(1,52) = 14.103, *p* < 0.001; Fig. 2b). Specifically, musicians significantly outperformed non-musicians on the music-in-noise task but not on the speech-in-noise task.

### Quasi-AP proficiency modulates perception of music, but not speech targets in noise

Next, we examined the effects of quasi-AP proficiency on HIN performance and assessed whether such quasi-AP effects differ depending on HIN task domain (music vs. speech). We expect quasi-AP abilities to enhance HIN performance, irrespective of whether the streams are speech or musical sounds. A 2 × 4 mixed ANOVA was performed on HIN performance accuracy with the factors AP proficiency (hiAP, medAP, loAP, NM) and task domain (music vs. speech). Figure 2c shows the proportion of correct HIN performance separated into MINT and SINT with the participants grouped by the level of AP proficiency. This analysis yielded a significant main effect of quasi-AP proficiency on HIN task performance (*F*(3,52) = 10.337, *p* < 0.001; Fig. 2c). HIN performance was significantly higher when the HIN task domain was music rather than speech (*F*(1,52) = 102.111, *p* < 0.001; Fig. 2c). A significant interaction between quasi-AP proficiency and HIN task domain was observed (*F*(3,52) = 9.289, *p* < 0.001; Fig. 2c). Specifically, quasi-AP proficiency modulates performance on the music-in-noise task, but has no effect on the speech-in-noise task. Post-hoc comparisons with Bonferroni correction (based on α/6 = 0.0083) on the level of quasi-AP proficiency showed that HIN performance accuracy differed significantly between the high-AP group and the med-AP group (*t*(13) = 3.954, *p* = 0.002), between the high-AP and the non-musician group (*t*(11) = 5.602, *p* < 0.001), between the med-AP and the non-musician group (*t*(11) = 2.844, *p* = 0.0081), and between the low-AP and the non-musician group (*t*(11) = 4.741, *p* = 0.005). All other pairwise comparisons were not significant.

### Quasi-AP proficiency modulates resilience to noise while perceiving melody in noise

**Figure 3:**
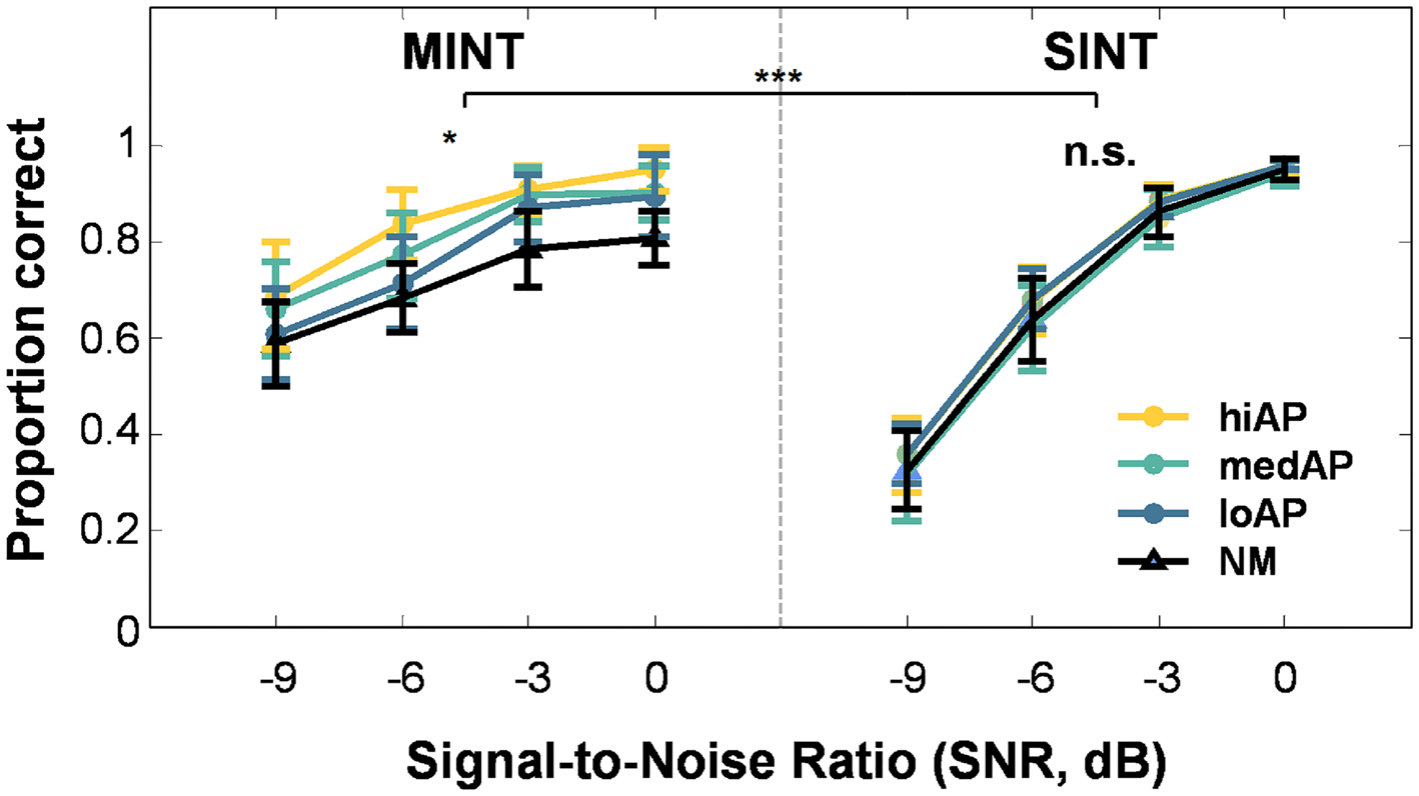
Mean performance accuracy on music-in-noise and speech-in-noise tasks as a function of SNR and quasi-AP proficiency level. Different-colored lines indicate different levels of quasi-AP ability. Error bars represents ± 1 SD. ***p < 0.001, **p < 0.01, *p < 0.05 level of significance; *ns* not significant.

Next, we assessed how the level of quasi-AP proficiency modulated HIN performance as a function of noise level depending on the task domain: music or speech. Figure 3 shows MINT and SINT performance as a function of SNR and AP-proficiency level. We ran a 2 × 4 × 4 mixed ANOVA on performance accuracy with the factors task domain (music vs. speech), quasi-AP proficiency (hiAP, medAP, loAP, NM), and SNR-level (0, − 3, − 6, − 9 dB). HIN performance was significantly better when listeners were required to detect a melodic target rather than a speech target (*F*(1,50) = 92.898, *p* < 0.001; Fig. 3). A significant main effect of quasi-AP proficiency on HIN performance was observed across all SNRs (*F*(3,50) = 9.610, *p* < 0.001; Fig. 3). Performance accuracy combined across both HIN task domains increased with increasing SNR level (*F*(3,150) = 994.258, *p* < 0.001; Fig. 3). The analysis also yielded a significant interaction between quasi-AP proficiency and the HIN task domain (*F*(3,150) = 8.678, *p* < 0.001; Fig. 3). Specifically, a higher level of quasi-AP proficiency was associated with enhanced performance for detecting melodic but not speech targets in noise. A significant interaction between SNR level and the HIN task domain was also observed (*F*(3,150) = 157.026, *p* < 0.001). There was no significant interaction between SNR level and quasi-AP proficiency level (*F*(9,150) = 0.612, *p* = 0.786). The analysis also revealed no significant three-way interaction (*F*(9,150) = 1.189, *p* = 0.306). This suggests that the quasi-AP enhancement effect, in addition to being domain-specific, was constant across SNR levels.

Post-hoc comparisons using Bonferroni corrections on different SNR conditions showed that performance accuracy differed significantly between all pairs (all *ps* < 0.001; see Supplementary Table S6). Post-hoc comparisons between different pairs of quasi-AP proficiency groups yielded significantly different performance accuracy between all pairs except for the comparison between the med-AP and the low-AP groups (all *ps* < 0.003; see Supplementary Table S7).

### Quasi-AP proficiency facilitates the use of spatial but not visual cues during segregation

**Figure 4:**
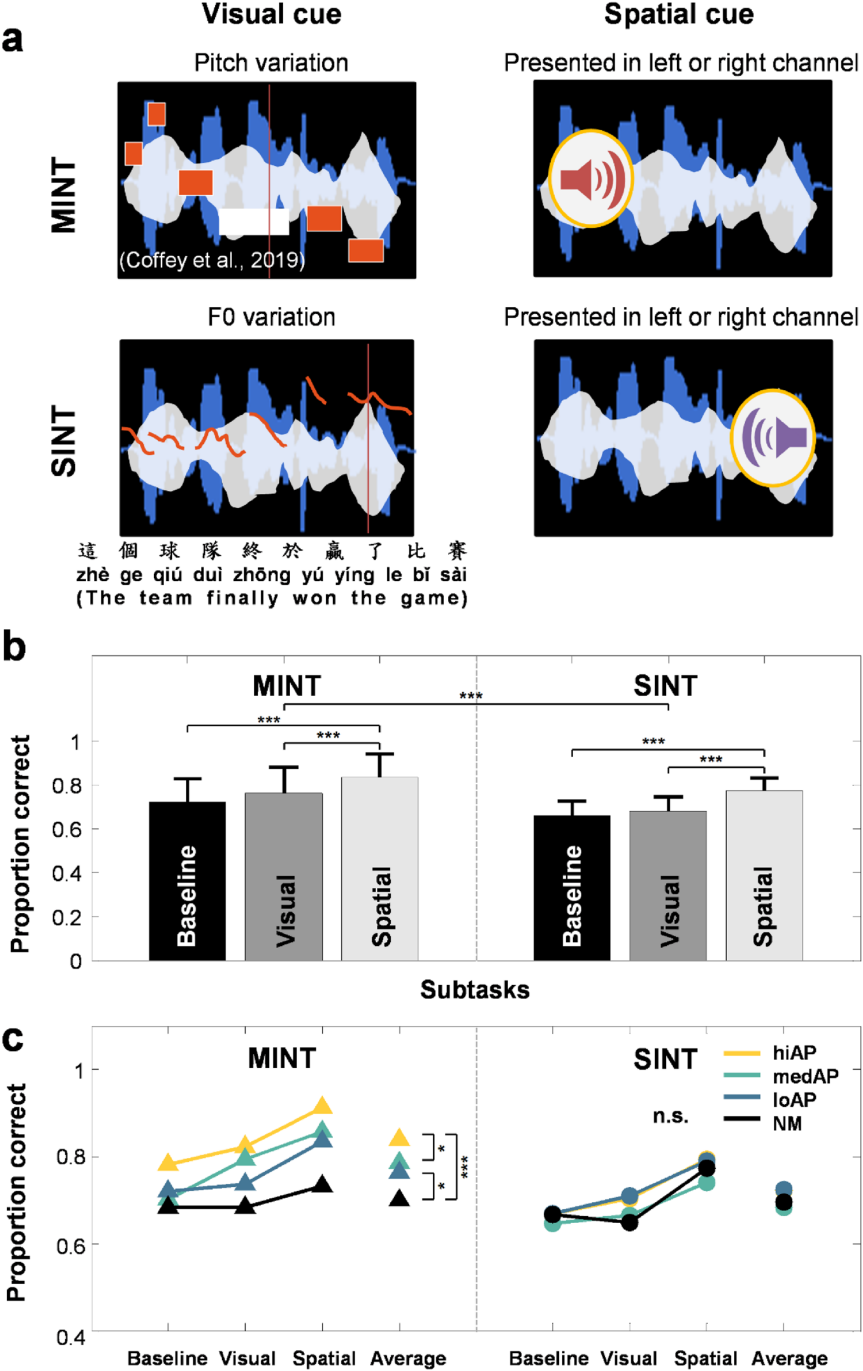
Performance accuracy on MINT and SINT subtasks. (**a**) Schematic depiction of the visual and spatial subtask for MINT (top panels) and SINT (bottom panels). Visual cue is represented as pitch variations (red) for melodic targets (blue) and F0 variations (red) for speech targets (blue). Spatial cue is represented as a speaker positioned to indicate left or right. Masking noise is represented in white. (**b**) Mean proportion correct for baseline, visual and spatial subtasks for MINT and SINT. (**c**) Mean proportion correct of the baseline, visual and spatial subtasks for MINT and SINT as a function of quasi-AP-proficiency groups. Error bars represents ± 1 SD. ***p < 0.001, **p < 0.01, *p < 0.05 level of significance; *n.s.* not significant.

An enhanced ability to use visuo-spatial cues has been reported to underlie the previously observed musician advantage in HIN perception, with some suggestions that visuo-spatial cues may increase streaming ability by drawing attention to target streams^19,40,41^. Hence, we examined whether quasi-AP proficiency might facilitate the use of visuo-spatial cues during HIN perception, and whether performance differs by HIN task domain. For the spatial cue condition, the sound level of the target and noise was adjusted to generate the perception of a target spatial location as either coming from the left or right side, with a small icon appearing on the screen before sound onset to indicate the target direction (Fig. 4a). For the visual cue condition, a graphic representation indicating either pitch height (of target melody) or the fundamental frequency contour (of target speech) was displayed concurrently with the target streams (Fig. 4a; see “Methods”). A 3 × 4 × 2 mixed ANOVA was performed on proportion correct with the factors subtask type (baseline, visual, spatial), quasi-AP proficiency level (hiAP, medAP, loAP, NM), and task domain (MINT vs. SINT). Listeners performed significantly better on MINT subtasks compared to SINT subtasks (*F*(1,50) = 62.338, *p* < 0.001; Fig. 4b). The analysis showed a significant main effect of subtask type on HIN performance (*F*(2,100) = 58.951, *p* < 0.001; Fig. 4b). There was a significant main effect of quasi-AP proficiency on HIN subtask performance (*F*(3,50) = 12.350, *p* < 0.001; Fig. 4c). Only one significant interaction was found between domain and quasi-AP proficiency level (*F*(3,50) = 5.895, *p* = 0.002; Fig. 4c). Specifically, quasi-AP proficiency facilitates subtask performance associated with music but not speech streams.

Post-hoc comparisons across subtask conditions revealed that HIN subtask performance was enhanced for the spatial subtask compared to the baseline condition (*t*(53) = 10.9, *p* < 0.001; Fig. 4b) but not with the addition of a visual cue (*t*(53) = 1.75, *p* = 0.258). The addition of a spatial cue also significantly facilitated HIN performance compared to the visual cue condition (*t*(53) = 8.9, *p* < 0.001). Post-hoc comparisons (Bonferroni corrected) across different quasi-AP proficiency groups revealed that the high-AP group outperformed the med-AP group on subtask performance (*t*(13) = 3.23, *p* = 0.003). Also, both the high-AP and low-AP groups performed better than the non-musicians across subtasks (hiAP vs. NM: *t*(11) = 5.247, *p* < 0.001; loAP vs. NM: *t*(11) = 2.855, *p* = 0.006).

### Auditory working memory of tonal but not linguistic materials is associated with music-in-noise performance

**Figure 5:**
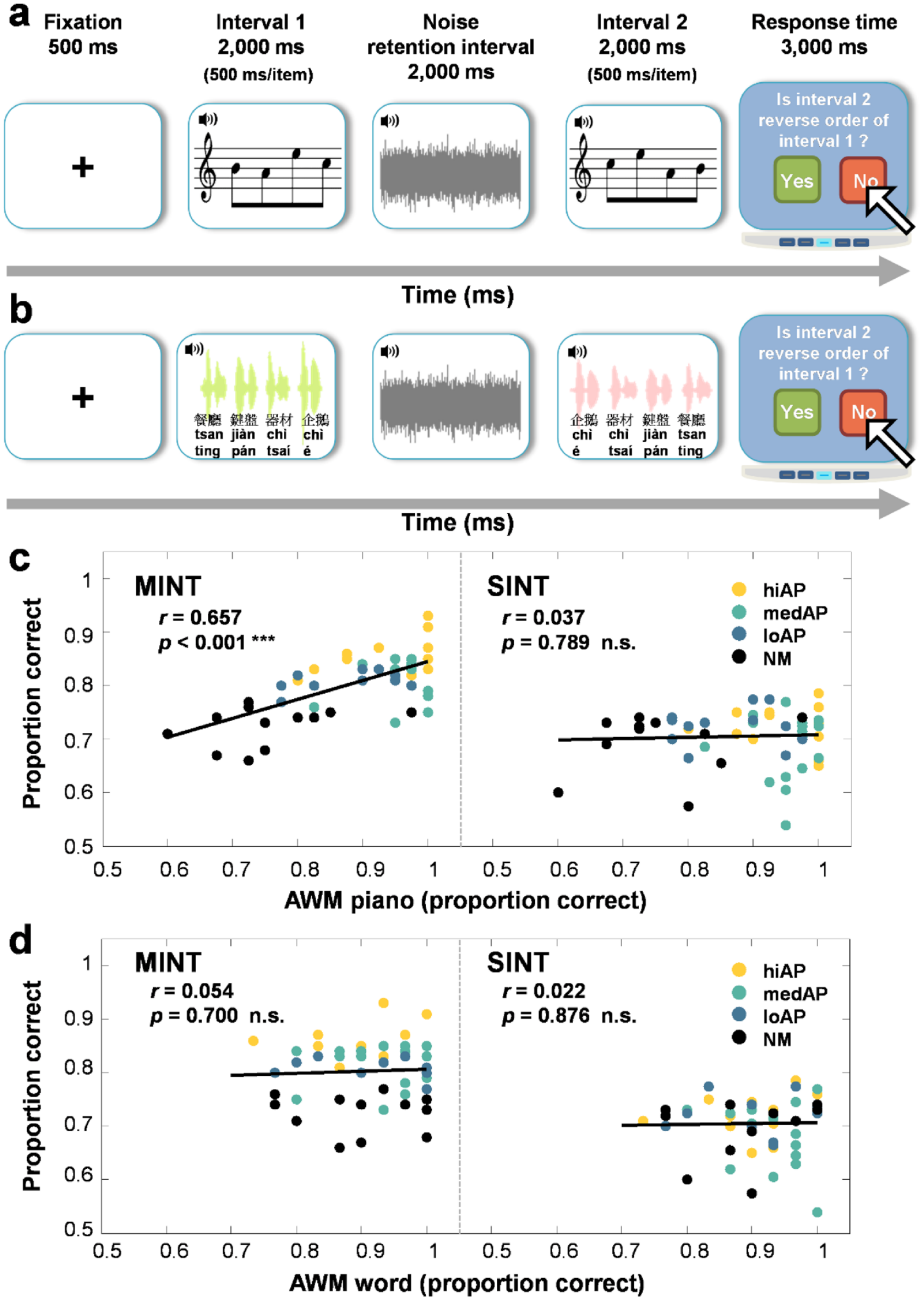
Correlations of the auditory working memory task and performance on MINT and SINT. Schematic diagram of the AWM task procedure for (**a**) piano tones and (**b**) Mandarin disyllabic compound words. Scatterplots depict correlation between (**c**) AWM task on piano tones with performance on MINT and SINT. (**d**) AWM task on words with performance on MINT and SINT. Different colored dots represent musicians with different quasi-AP proficiency levels.

Some studies suggest that superior AWM performance correlates with better SIN performance^19,38^; thus, we also tested the relationship between AWM abilities and HIN performance. Previous assessment of AWM abilities has mostly used linguistic measures (phonological/digit span); thus, we examined the relevance of linguistic and non-linguistic (tonal) AWM information to HIN perception. AWM was examined using a reversed sequence judgment task on both piano tones^42^ and Mandarin disyllabic compound words, which required a listener to judge whether a second four-tone (or word) sequence was presented in the time-reversed order of the first sequence (Fig. 5a,b). Correlational analyses between tonal/speech AWM ability and musical/linguistic HIN performance revealed a positive association between AWM for piano tones and performance on music-in-noise task (*r* = 0.657, *p* < 0.001; Fig. 5c), but not speech-in-noise task (*r* = 0.037, *p* = 0.789; Fig. 5c). On the other hand, AWM for words was not correlated with performance on HIN tasks with either melodic or speech targets (MINT: *r* = 0.054, *p* = 0.700; SINT: *r* = 0.022, *p* = 0.876; Fig. 5d). The results showed that only tonal AWM abilities was associated with music-in-noise perception. These findings indicate that the domain-specific effect of quasi-AP abilities on HIN processing extended to AWM abilities. Better tonal AWM abilities were associated with benefits for perceiving music but not speech in noise, which in turn was modulated by quasi-AP proficiency (for analysis on AWM vs. quasi-AP proficiency see Participants’ Characteristics).

### Relationships to musical training

**Figure 6:**
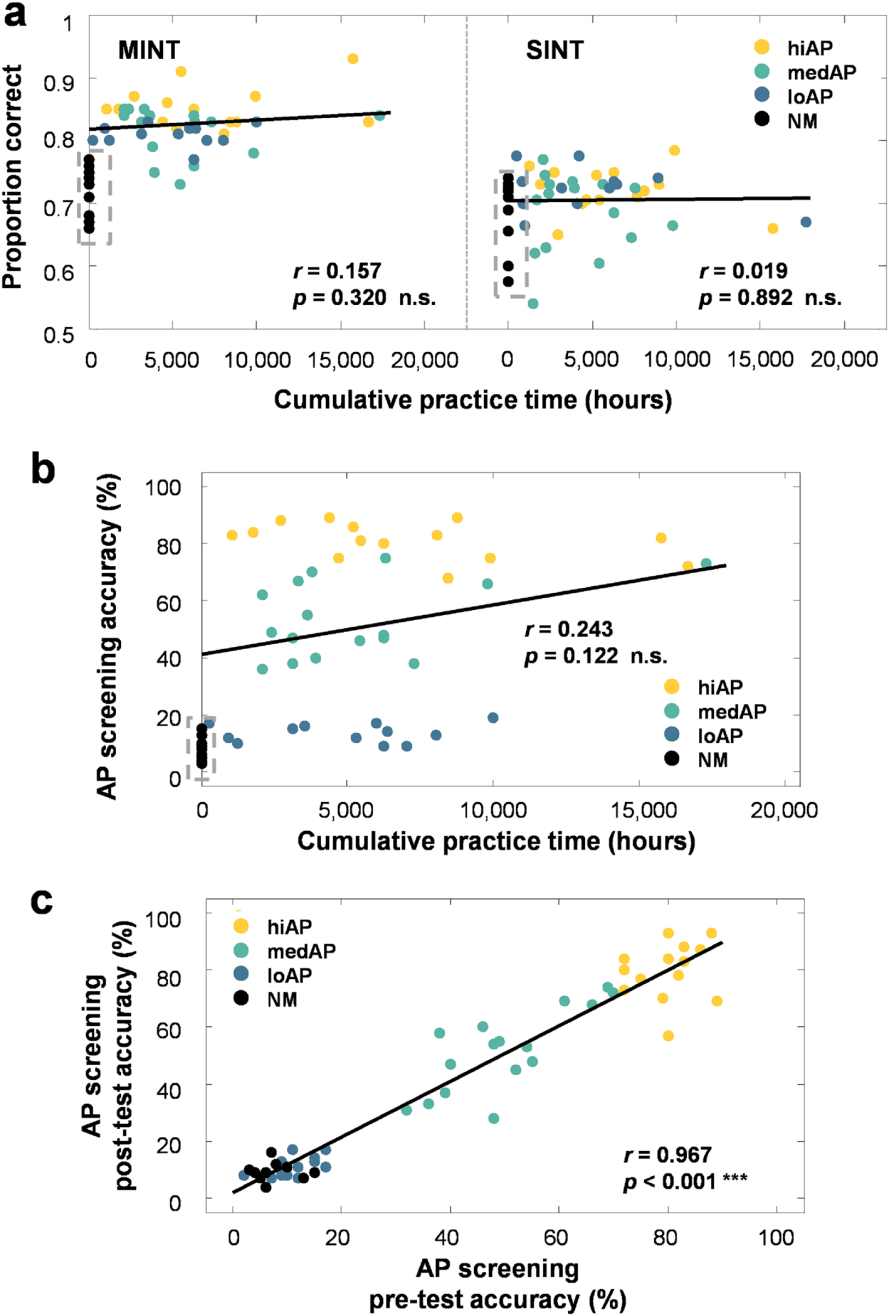
Correlations of cumulative practice hours and screening scores on MINT and SINT performance. (**a**) Scatterplots depict correlation between cumulative practice hours (computed from MMHQ) for music-in-noise task (left panel) and speech-in-noise task (right panel). (**b**) Correlation between AP screening score and cumulative practice hours. Note that non-musicians (black dots) were excluded from this analysis due to non-applicable training hours. (**c**) Correlation between AP screening score for pre and post-testing session. Each dot represents individual subject with quasi-AP proficiency level coded by different color.

Musical training experience is sometimes linked to better SIN perception^15^; thus, we tested the relationship between musical training hours and HIN performance. Music training hours were the accumulated total practice hours of the individual (information computed from the Montreal Music History Questionnaire (MMHQ); see “Methods”). For this analysis, only the group of musicians was considered, since practice hours were in general not applicable to the non-musicians (i.e., we excluded compulsory music classes in school as training hours). No significant correlation was found between musical training hours and the perception of melody in noise (*r* = 0.157, *p* = 0.320; Fig. 6a), or for the perception of speech in noise (*r* = 0.019, *p* = 0.892; Fig. 6a). We also examined the relationship between musical training hours and quasi-AP abilities and obtained no significant correlation between these two measures (*r* = 0.243, *p* = 0.122; Fig. 6b). Lastly, to confirm the stability of AP proficiency level, we ran a correlation analysis between the same participant’s quasi-AP proficiency screening score prior to and after completing all the testing sessions. This analysis revealed a significant positive correlation between the pre- and post- AP screening scores (*r* = 0.967, *p* < 0.001; Fig. 6c), indicating that the level of quasi-AP proficiency was a relatively stable trait.

## Discussion

For the first time, we report a quasi-AP modulatory effect on hearing-in-noise perception that is domain-specific. In particular, when the target to be detected consists of melodic streams, a quasi-AP enhancement effect is observed on HIN perception. In contrast, no quasi-AP enhancement effect is observed for perceiving speech streams embedded in noise backgrounds. Importantly, this is the first study to demonstrate that the level of quasi-AP proficiency, considered as a general ability that varies across the spectrum, can modulate HIN perception in a domain-specific manner. Contrary to previous studies, which mostly restricted AP ability to a limited range within 4% to 20% of the population^43,44^, here we studied AP proficiency by comparing listeners with different intermediate levels of AP proficiency. We also observed a domain-specific effect of quasi-AP advantage on HIN performance: quasi-AP ability is associated with improved perception of music in noisy backgrounds, but not of speech. While performance in detecting either speech or music targets in noise improved with increasing SNR level, the quasi-AP enhancement remained constant across all noise levels. Additionally, comparison across the various HIN subtasks with concurrent presentation of visuo-spatial cues showed that the addition of spatial cues, but not visual cues enhanced perception in noise. Interestingly, the quasi-AP advantage was only observed for spatially-presented melodic streams, not speech streams, suggesting a domain-specific quasi-AP advantage in the use of spatial cues during segregation. The domain-specific quasi-AP effects on HIN perception were also corroborated by correlations between other AP proficiency verification measures (i.e., pitch adjustment, post screening), suggesting a pronounced quasi-AP advantage associated with segregating music materials.

Importantly, we showed the effect of quasi-AP enhancement on MIN perception by comparing listeners with different degrees (high, medium, low) of quasi-AP abilities. The quasi-AP advantage is consistent with previous studies demonstrating a musician advantage in segregating a target melody from a “noise background” consisting of multiple melodies^6,19^. Further, here we provide evidence that the musician advantage in MIN perception is modulated by the level of quasi-AP proficiency. Importantly, the quasi-AP enhancement remained when tonal AWM performance was included as a covariate in the analysis (*F*(3,49) = 14.058, *p* < 0.001). The significant quasi-AP effect after removing AWM, in addition to the lack of correlation observed with training hours (Fig. 6b), substantiate the finding of a quasi-AP advantage in perceiving music in noise. Certainly, musicians can differ in various aspects, but AP ability is one of the few characteristic traits that is known to both be genetic and require a critical period during development. It is also relatively immune to training^33^. To validate its stability level, we also performed a post-screening for AP ability for all the listeners (i.e., the post-screening occurred more than 6 months later for some listeners due to the Covid 19 pandemic situation) and obtained a high correlation (see Fig. 6c).

In contrast to perceiving musical targets, we did not find a modulatory effect of quasi-AP ability on speech encoding in noise. This lack of musician advantage in SIN perception, while consistent with several studies that have reported no musician advantage in identifying speech under different types of noise^20^, talkers^45^, or spatial locations^13,22,23^, is also in contradiction to other reports^9,11,13–16^. One reason may be the different kinds of task materials that affect SIN results^7^, as complete sentences of tonal (Mandarin) language were used in this study, while previous studies mostly used syllables (e.g., /ba/, /ma/, /da/)^11,15,17,25^, non-tonal (e.g., English, French) meaningful words or sentences^1,13,14,20^, or syntactically correct sentences without semantic or contextual cues^18,24^. The differences between languages (tonal vs. non-tonal) and type of sentences may have caused the discrepant findings observed on SIN performance. In relation to this, meaningful speech (used here) may cause high-level processing like top-down semantic perception. Native speakers potentially drive the same level of top-down benefits from contextual cues for speech perception, resulting in similar level of accuracy across all listeners on SIN tasks, independent of quasi-AP proficiency levels. Further, given that the musician advantage on SIN performance may be differentially affected by masker type^7,46^, we applied another type of noise masker, multi-talker babble with four talkers, to introduce more effective masking (via informational masking vs. peripheral as in speech-shaped noise)^46^ to examine whether a quasi-AP effect could be observed. Similarly, no quasi-AP enhancement effect on SIN processing was observed, suggesting that perceiving speech in noise may not rely on quasi-AP abilities (Supplementary Fig. S2).

Interestingly, our results suggest a quasi-AP advantage on the use of additional cognitive cues during HIN perception that is also contingent on the domain (music vs. speech) of the auditory stream. Listeners possessing higher levels of quasi-AP ability benefit more from the use of additional cues only when segregating melodic streams, while no such cue advantage was observed with speech streams. In particular, we found the cue enhancement of HIN performance only for spatial cues and not for visual cues, contrary to Coffey et al.^19^ who reported a cue enhancement effect for music-in-noise perception with both spatially and visually presented streams. Most importantly, we revealed that quasi-AP proficiency modulates the use of spatial cues for perceiving melodic targets in noise, but the presentation of visual (musical or F0) pitch cues during stream segregation is not quasi-AP dependent. Our finding that spatially presented cues better facilitate HIN perception (here using interaural-level difference, ILD) was consistent with studies reporting better HIN perception when the target and noise streams were presented with a spatial location generated with ITD and/or ILD cues^47,48^ or with a more naturalistic setting either simulated or using loudspeaker arrangements^17,18^. One potential reason for the lack of visual cue enhancement effect could be that, in order for a visual cue to successfully facilitate auditory processing, it should both predict in form and occur early in time^40,41^. Here, we presented the visual pitch/F0 cues simultaneously with the melodic and speech streams, and thus could render the cue predictor value less pronounced. Future studies could manipulate the relative timing of occurrence between the visual cue and auditory speech or melodic streams to examine its effects on perception against a noisy background.

In terms of cognitive factors affecting HIN performance, we found AWM ability to exhibit a similar domain-specific advantage, which is also modulated by quasi-AP proficiency. Better tonal WM ability (i.e., piano tones) was associated with an enhanced perception of music but not speech in noise. In contrast, better linguistic AWM ability (i.e., Mandarin disyllabic words) was not associated with improved HIN perception for both domains. A recent meta-analysis has revealed a moderate association (*r* = 0.28) between AWM and speech-in-noise abilities, with most studies using phonological WM or digit span measures^7^. While we expected AWM abilities to correlate with SIN performance, only the abilities to recall tonal information were found to be associated with MIN perception. The discrepancies between studies suggest that the nature of the WM elements may affect HIN performance. In particular, one recent study has reported that working memory for frequency (pure tones), rather than phonological WM, facilitates speech perception in noise^38^. The idea that non-linguistic WM precision is more relevant to SIN perception is consistent with the current association observed between tonal WM and MIN perception. In addition, the domain-specific AWM effects on HIN perception observed here corroborated previous reports proposing two differing neural networks underlying verbal and tonal WM in musicians^49,50^. Further, we demonstrated that superior AWM performance observed in musicians could be modulated by quasi-AP ability, albeit specific to tonal materials. The quasi-AP effect on AWM is consistent with the proposed notion of quasi-AP as a form of pitch memory, in that quasi-AP listeners may form reference tones from a familiar song or musical instrument^37,51^. In line with this, some studies have reported quasi-AP musicians to exhibit more extensive activation of a right hemisphere network for maintenance of pitch in AWM (i.e., the superior and middle temporal gyri and the right dorsolateral prefrontal cortex) during pitch naming^52^.

Lastly, while we attempted to control for the potential mediating effects of relative pitch ability, it was nevertheless correlated with absolute pitch proficiency and partially limited our explanation of quasi-AP proficiency as the only mediating factor for musician advantage in MIN performance. One potential explanation could be that we deliberately chose a relative pitch task design that did not require explicit naming of the RP intervals, so that people without music training could also complete the task without problems^53^. As such, the task could be relatively easy for listeners with musical training, hence the observed enhanced performance. On the other hand, the observation that listeners with higher quasi-AP proficiency also performed better on the RP task is consistent with some studies that reported AP possessors to also exhibit enhanced performance on RP tasks (although the reverse has also been reported^54^). Nevertheless, the fact that RP ability can be trained and improved much more easily than AP suggests that RP ability could very likely vary over time, compared to AP ability.

In summary, we report a domain-specific form of HIN perception that is modulated by the proficiency of quasi-AP ability across the general population. Better comprehension of melodic targets under challenging listening conditions is associated with higher quasi-AP proficiency, while speech comprehension is not. Listeners possessing higher levels of quasi-AP proficiency benefit more from the facilitating effect of concurrent spatial cues during MIN perception, whereas visual cues are not modulated by quasi-AP ability during stream segregation. The mediating factor of auditory working memory also exhibits a domain-specific modulatory effect with only working memory for tonal materials associated with better HIN perception. These findings suggest that quasi-AP ability is an important modulating factor for perceiving music under challenging listening environments.

## Methods

### Participants

Forty-two musicians and 12 non-musicians aged 19 to 26 years old (mean ± SD = 20.91 ± 1.19 years, 29 males) participated. All participants had normal hearing measured using a standard audiometric procedure (< 20 dB HL thresholds at octave frequencies between 125 and 8000 Hz; MA 25e; Maico Diagnostics, Minneapolis, USA). All participants were native speakers of Mandarin Chinese, and all but four were right-handed as identified using the Edinburgh Handedness Inventory^55^. The participants reported no history of neurological or psychiatric disease. Most of the participants were students at National Central University or enrolled in the music department at nearby universities. For the musician group, qualifying participants were required to have commenced formal music training before the age of six (mean age = 5.61, SD = 1.85) and continued to take formal music lessons for more than 7 years (> 7 years of musical activity and > 4000 h of cumulative lifetime practice). Qualifying musicians were required to be currently playing a musical instrument for at least 5 h a week. The primary instrument for all qualified musicians was piano. The musician group was further defined into three groups according to their score on AP screening (details in Table 1). The non-musician group was defined as individuals with less than 1 year of experience with formal music lessons (except for compulsory music courses in school). Written informed-consent was obtained from each participant in accordance with the Institutional Review Board at National Taiwan University, Taiwan. All procedures were approved and performed in accordance with the guidelines of the Institutional Review Board at National Taiwan University, Taiwan.

### Auditory stimuli generation and apparatus

All testing was conducted in a double-walled, acoustically isolated steel chamber (interior dimensions, 2.5 m × 2.5 m × 2 m; Industrial Acoustics Company). Stimuli were generated using MATLAB (2015b, MathWorks, USA) on an ASUS Vento PC and presented at a sampling rate of 44.1 kHz through 16-bit digital-to-analog converters (Creative Sound Blaster X-Fi Titanium). Sounds were presented through Sennheiser headphones (HD380 Pro, Wedemark, Germany) at 70 dB SPA.

### Testing procedure

The testing procedure consisted of four sessions. In the first session, participants filled out the full version of the Montreal Music History Questionnaire (MMHQ) online (Mandarin Chinese version^56^), which assessed their demographic background and detailed information regarding music training. Participants were then assessed for absolute pitch proficiency in the lab using an in-house pitch identification screening consisting of 50 sine-wave pure tones and 50 piano notes randomly selected from five octaves ranging from C2 to B6 (65.4–1975.5 Hz; A4 = 440.0 Hz). Participants who qualified as musicians were divided into three AP groups based on their screening accuracy performance: high-AP (hiAP; > 80%), medium-AP (medAP; 33 − 80%), and low-AP group (loAP; 8 − 33%). Participants in the non-musician group had an average screening score of less than 8% accuracy (i.e., below-chance performance). The first session took approximately one hour to complete. Figure 1a displays the screening results and criteria for division of the AP proficiency groups. Chance level was calculated from the reciprocal of the total number of AP screening note-naming response alternatives, i.e., p = 1/(12 musical-note names, C, C#, D, D#, etc.) = 0.083 or 8.3%.

The next three testing sessions (sessions 2–4) each lasted approximately 80 min. In the second session, participants completed the Music-in-Noise Task (MINT)^19^, which consisted of five subtasks. In the third session, participants completed the Mandarin version of the Hearing-In-Noise Test (M-HINT)^57^ consisting of four subtasks. In the last session, participants completed a relative pitch (RP) task, followed by an assessment of auditory working memory (AWM) for tones and words, and a fine pitch adjustment task. Lastly, a post-screening for absolute pitch ability was administered again to confirm the stability of AP proficiency level. The four sessions were implemented on four separate days over no more than six months. The order at which the four sessions (except for the pre-screening session) was implemented was randomized across participants. With the exception of the post-screening task, all other tasks (i.e., RP, AWM, pitch adjustment) implemented within the session were also run in a randomized order across participants.

### Experiment materials and task design

#### AP screening task

An in-house AP screening task was used to screen for AP proficiency level prior to and after testing sessions^58^. The stimuli used for screening of AP ability consisted of 50 pure tones and 50 piano notes run in two separate blocks of 50 trials each. Each tone was 1000 ms in duration, with 100 ms rise-decay ramps. On each run, 50 pure tones were randomly chosen from musical note frequencies in the range C2 to B6 (65.4–1975.5 Hz based on an equal-tempered scale; A4 = 440.0 Hz). The notes were randomly selected in each trial with the constraint that two successive notes were at least 2 octaves ± 1 semitone apart^29^. A 600 ms burst of white Gaussian noise was introduced 600 ms after termination of each stimulus, followed by 1200 ms of silence during which subjects responded. The response time limit of 1200 ms was enforced to restrict the potential use of relative strategies during AP identification. The noise was introduced to reduce echoic (sensory) trace memory cues. Participants were asked to name each note by its musical note name (e.g., Do/Fa#). In the piano condition, piano notes were randomly presented to participants from the same range (C2 to B6) and with the same constraint as in the pure-tone condition in a 50-trial run. Scoring of responses was similar to that used by Baharloo et al.^29^. Participants received 1 point for correct identification and 0.5 point for identification within a semitone of the correct note (i.e., judging C# as C). We used a slightly more stringent criterion than Baharloo et al.^29^, who score 1-semitone errors as 0.75 points. Octave designation was excluded from scoring since previous studies showed no significant difference between AP and non-AP subjects in identifying octaves^59^. Chance performance was 4.15 points (total score = 50, equivalence of 8.33%).

#### MINT

The MINT, developed by Coffey et al.,^19^ was used to assess the ability to segregate a target melody from background noise consisting of a mixture of four melodic streams. The MINT consisted of five different subtasks: baseline, rhythm, visual, spatial, and prediction using a match–mismatch design (Fig. 2a). In each trial, the participant heard a short instrumental music excerpt, followed by an instrumental music excerpt presented in noise composed of multi-music materials. The participant’s task was to indicate whether the contents of the two music excerpts was the same or different with a key press response. For the rhythm condition, the melody was of identical pitches except that the rhythm was the only distinguishing feature between the two. For the visual and spatial subtasks, an additional visual or spatial cue was appended onto the music excerpts (Fig. 4a). Finally, for the prediction task, the music excerpt was first presented in silence, which helped the participant to anticipate the coming target signal when embedded in noise. With the exception of the prediction block, the melody-plus-noise stream always occurred before the melody-alone stream. For all of the five subtasks, four different signal-to-noise ratios were employed (− 9, − 6, − 3, and 0 dB) to assess performance. The level of background noise was kept constant at 65 dB, while the level of the target melody was lowered according to specific SNR. Each participant completed a total of five subtask blocks of 20 trials each in a randomized order.

#### SINT

To assess speech-in-noise performance, target sentences were taken from the M-HINT^57^. The M-HINT corpus includes a total of 240 Mandarin sentences spoken by a male speaker, with each sentence consisting of a total of 10 words and lasting approximately 2.5 s on average. For the baseline SINT task, a total of 30 sentences were randomly selected without replacement from the M-HINT sentence corpus for each SNR condition. Four different SNR levels (0, − 3, − 6, and − 9 dB) were assessed by manipulating the level of the target sentence while keeping the level of masking noise constant at 65 dB. The selection of these 4 SNR levels was empirically determined in a pilot study to ensure that these levels captured the range of performance while avoiding the ceiling effect for normal-hearing Mandarin young adults. In addition, these SNR levels could be compared with performance in the MINT conditions. Two types of background noise maskers were examined. The masker was either a speech-shaped noise, a Gaussian noise modulated by the average spectrum shape of the target speech, or a multi-talker babble masker consisting of four talkers (two males, two females). Each participant completed a total of eight blocks (30 sentences × 4 SNRs × 2 maskers) in a randomized order. Participants were instructed to type out the they sentence heard into a textbox presented as a GUI on screen.

To compare the effects of visual and spatial cues on HIN performance with speech vs. melody targets, we further manipulated visual and spatial cues during the SINT task according to the visual/spatial blocks of MINT^19^. For the visual cue condition, Praat (version 6.1.16)^60^ was used to extract the F0 contour for each sentence in the M-HINT set and output the text file. For visual presentation, the comet function (MATLAB) was used to simultaneously display the F0 contour of the target sentence on the screen while the sentence was presented auditorily. The rate of F0 contour presentation was adjusted to match the sentence presentation rate. For the spatial subtask condition, the sentence was presented from either the left or right channel alone with a visual “speaker” cue indicating the spatial location to the participant on the screen (Fig. 4a). The choice of the left and right channel was randomized on a trial-by-trial basis. The noise masker was presented from both channels. The procedures for visual and spatial SINT subtasks were identical to those of the baseline task. Each participant completed a total of 240 sentences (30 sentences × 4 SNRs × 2 maskers) in a randomized order per spatial/visual cue condition. No feedback was given at any point during the experiment. Speech intelligibility was computed as the proportion of words typed correctly using a customized MATLAB script written in the P.I.’s lab to automatically compare the sentences word by word. Words that were obviously mistyped as homophones were considered correct.

#### AWM task

The AWM task paradigm was adapted and modified from the procedure employed by Albouy et al.,^42^. The task involved identifying whether two four-tone sequences (S1, S2), separated by a retention interval of 2 s of broadband noise, were presented in reversed order. Each four-tone sequence was composed of four randomly selected 500-ms pure tones each with different pitch height randomly selected within a range from C3 to B5 (130.81–987.77 Hz). Tones were selected without replacement and were presented successively with an inter-stimulus-interval (ISI) of 0 ms. The standard sequence (S1) had 120 different combinations of four-tone melodies, and the comparison sequence (S2) had the same melody except that it was time-reversed in half of the trials. For the other-half of the trials, the comparison sequence S2 had the same four tones but in a randomized order. The participant’s task was to determine whether the second sequence was presented in the time-reversed order of the first sequence by pressing one of two keys on the keyboard.

An equivalent AWM task using four Chinese compound word sequences was also implemented. The procedure was identical to that of the musical-note sequences except that each tone was replaced by a Chinese disyllabic word. A total of 120 Chinese disyllabic words (nouns) were selected from the Academia Sinica Balanced Corpus of Modern Chinese, which consists of meaningful typical nouns used in Mandarin Chinese. To control for the difficulty level, the frequency of these disyllabic words was equated across trials according to the frequency of occurrence in Mandarin Chinese as measured by the Accumulated Word Frequency count tool provided by Academia Sinica (https://elearning.ling.sinica.edu.tw/CWordfreq.html). Text to speech software was used to output the words into audio files of a male speaker. These audio files were then digitally processed so that each audio item lasted 500 ms. The variation in the sound level of each word in the audio files was normalized. The procedure was identical to the one used for the tone AWM task. Each participant completed two blocks of 60 trials each for the melodic or word sequence condition. Figure 5a,b display the schematic procedure for the AWM task for both the tonal and word conditions.

#### RP task

Relative pitch ability was assessed using a three-interval forced choice design via GUI similar to the procedure used by Hove et al.^53^. The stimuli consisted of piano tones randomly selected from the tonal scale within two sharp/flat notations based on Western music conventions: G-major, F-major, E-minor, and D-minor scales in the 3–5th octave range. The musical intervals were randomly selected from musical intervals comprising major thirds, perfect fifth, and minor seventh. On each trial, one musical interval was randomly selected and presented twice sequentially with an ISI of 300 ms. All tones were 500 ms in duration, with 20 ms linear rise-decay ramps. All intervals consisted of ascending melodic intervals, in which the second tone of each interval was always higher in frequency than the first. The intervals were selected with the constraints that intervals for adjacent trials were from two different tonal scales and differed by more than 2 semitones. The participant’s task was to identify the interval presented by choosing one of three buttons on the screen labeled “3,” “5,” or “7,” with the number indicating the musical interval corresponding to major thirds, perfect fifth, and minor seventh, respectively. Each participant was allowed to practice for 12 trials at most with feedback before completing the test session of 24 trials. No feedback was given at any time during the actual test.

#### Pitch adjustment task (PAT)

A PAT was used to quantify fine-grain differences in absolute pitch proficiency^6,58^. The task was to adjust the frequency of a pure tone within a restricted time interval of 5 s to match a visually presented musical-note target (i.e., Do [C], La [A], etc.) selected from a set of 60 musical-note frequencies across five octaves (C2 to B6; same as screening). In each trial, one of 12 notes was randomly selected and displayed as text on screen. The participant adjusted an unlabeled GUI slider to change the stimulus frequency and pressed a button to hear the stimulus. The slider range was kept constant at 3/4 octave but was randomly positioned in each trial with respect to the target frequency. A 3/4 octave range was used instead of a full octave to ensure a single solution. The two step sizes of the slider were set at 10% and 1% of the total range with a resolution of 1% (0.09 semitone). During the adjustment interval, participants were able to press the target button on the GUI monitor to hear the 1-s target pitch as many times as desired. Once a final adjustment was made, the subject pressed a button to record the results. The slider was then reset to the middle position at the beginning of each trial. A total of three adjustment sessions were run for each of 12 musical notes.

### Statistical procedures

All statistical analyses were performed using MATLAB (2015a, MathWorks, USA) and SPSS 18.0. (IBM). An alpha level of p < 0.05 (two-tailed) was set a priori to determine statistical significance. Standard parametric tests were performed at the group level (mixed ANOVAs, paired sample *t*-tests, Pearson correlations). Multiple comparisons were corrected using Bonferroni corrections (family-wise α = 0.05).

## Supplementary Information

Supplementary Information.

## Data Availability

All data are available from the corresponding author upon reasonable request and with permission from the Research Ethics Committee of National Taiwan University.
