# Supplementary material for: Domain-specific hearing-in-noise performance is associated with absolute pitch proficiency
Source: Sci Rep. 2022 Sep 29;12:16344. doi: 10.1038/s41598-022-20869-2 (PMC9521875; doi:10.1038/s41598-022-20869-2)
Supplement: Supplementary file 1 — Supplementary Information. [file 41598_2022_20869_MOESM1_ESM.docx]

**Supplementary Table 1. Summary of cutoff criteria used for AP proficiency groups based on note identification**

| **Research study** | **Number of groups** | **AP Group division** | **Cutoff criteria**  **(percent accuracy)** |
| --- | --- | --- | --- |
| Wilson et al. (2009) | 3 | AP vs.  quasi-AP vs.  non-AP(RP) | > 90%  20−90%  < 20% |
| McLachlan et al. (2013) | 3 | AP vs.  quasi-AP vs.  non-AP(RP) | 80−100%  20−70%  0−14% |
| Schuze et al. (2013) | 2 | AP vs. non-AP | 50% approx. |
| Burnham et al. (2014) | 2 | AP vs. non-AP | 90% |
| Leite et al. (2016) | 3 | Conservative-AP vs.  Inclusive-AP vs.  non-AP | > 85%  20−80%  < 8.3% |
| Wenhart et al. (2019) | 2 | AP vs. non-AP(RP) | 33% |
| Rogenmoser et al. (2021) | 2 | AP vs. non-AP | 30% approx. |

**Supplementary Table 2. Post-hoc comparisons across the four AP-proficiency groups on AP screening score**

| Bonferroni-adjusted statistics | | | | | | |
| --- | --- | --- | --- | --- | --- | --- |
| Condition | *t* | | | *df* | *p-value two-tailed*  *(based on α/6 = 0.0083)* | |
| high-AP vs. med-AP | | 10.049 | 13 | | | < .001*** |
| high-AP vs. low-AP | | 21.502 | 11 | | | < .001*** |
| high-AP vs. non-musician | | 22.180 | 11 | | | < .001*** |
| med-AP vs. low-AP | | 12.521 | 11 | | | < .001*** |
| med-AP vs. non-musician | | 13.219 | 11 | | | < .001*** |
| med-AP vs. non-musician | | 0.653 | 11 | | | .167 |

**Supplementary Table 3**. **Post-hoc comparisons across the four AP-proficiency groups on pitch adjustment accuracy (deviation in semitone)**

| Bonferroni-adjusted statistics | | | | | | |
| --- | --- | --- | --- | --- | --- | --- |
| Condition | *t* | | *df* | | *p-value two-tailed*  *(based on α/6 = 0.0083)* | |
| high-AP vs. med-AP | | −1.616 | | 13 | | .112 |
| high-AP vs. low-AP | | −9.094 | | 11 | | < .001*** |
| high-AP vs. non-musician | | −9.105 | | 11 | | < .001*** |
| med-AP vs. low-AP | | −7.820 | | 11 | | < .001*** |
| med-AP vs. non-musician | | −7.831 | | 11 | | < .001*** |
| low-AP vs. non-musician | | −0.011 | | 11 | | .167 |

**Supplementary Table 4**. **Post-hoc comparisons across the four AP-proficiency groups on AWM recall accuracy on tonal materials**

| Bonferroni-adjusted statistics | | | | | | |
| --- | --- | --- | --- | --- | --- | --- |
| Condition | *t* | | | *df* | *p-value two-tailed*  *(based on α/6 = 0.0083)* | |
| high-AP vs. med-AP | | −0.591 | 13 | | | .167 |
| high-AP vs. low-AP | | 2.539 | 11 | | | .014 |
| high-AP vs. non-musician | | 6.225 | 11 | | | < .001*** |
| med-AP vs. low-AP | | 3.183 | 11 | | | .002** |
| med-AP vs. non-musician | | 6.980 | 11 | | | < .001*** |
| low-AP vs. non-musician | | 3.551 | 11 | | | .001** |

**Supplementary Table 5**. **Post-hoc comparisons across the four AP-proficiency groups on relative pitch task accuracy**

| Bonferroni-adjusted statistics | | | | | | |
| --- | --- | --- | --- | --- | --- | --- |
| Condition | *t* | | | *df* | *p-value two-tailed*  *(based on α/6 = 0.0083)* | |
| high-AP vs. med-AP | | 1.528 | 13 | | | .135 |
| high-AP vs. low-AP | | 2.898 | 11 | | | .006* |
| high-AP vs. non-musician | | 9.655 | 11 | | | < .001*** |
| med-AP vs. low-AP | | 1.448 | 11 | | | .156 |
| med-AP vs. non-musician | | 8.205 | 11 | | | < .001*** |
| med-AP vs. non-musician | | 6.443 | 11 | | | < .001*** |

**Supplementary Table 6. Post-hoc comparisons between different SNR levels on HIN performance accuracy**

| Bonferroni-adjusted statistics | | | |
| --- | --- | --- | --- |
| Condition | *t* | *df* | *p-value two-tailed*  *(based on α/6 = 0.0083)* |
| SNR = −9 vs. −6 | −22.000 | 53 | < .001*** |
| SNR = −9 vs. −3 | −38.300 | 53 | < .001*** |
| SNR = −9 vs. 0 | −48.444 | 53 | < .001*** |
| SNR = −6 vs. −3 | −18.000 | 53 | < .001*** |
| SNR= −6 vs. 0 | −27.000 | 53 | < .001*** |
| SNR = −3 vs. 0 | −9.000 | 53 | < .001*** |

**Supplementary Table 7. Post-hoc comparisons between pairs of AP-proficiency groups on HIN performance accuracy**

| Bonferroni-adjusted statistics | | | | | | |
| --- | --- | --- | --- | --- | --- | --- |
| Condition | *t* | | | *df* | *p-value two-tailed*  *(based on α/6 = 0.0083)* | |
| high-AP vs. med-AP | | 3.000 | 13 | | | .001** |
| high-AP vs. low-AP | | 2.429 | 11 | | | .003* |
| high-AP vs. non-musician | | 5.286 | 11 | | | < .001*** |
| med-AP vs. low-AP | | −0.385 | 11 | | | .120 |
| med-AP vs. non-musician | | 2.692 | 11 | | | .002** |
| low-AP vs. non-musician | | 2.857 | 11 | | | .001** |


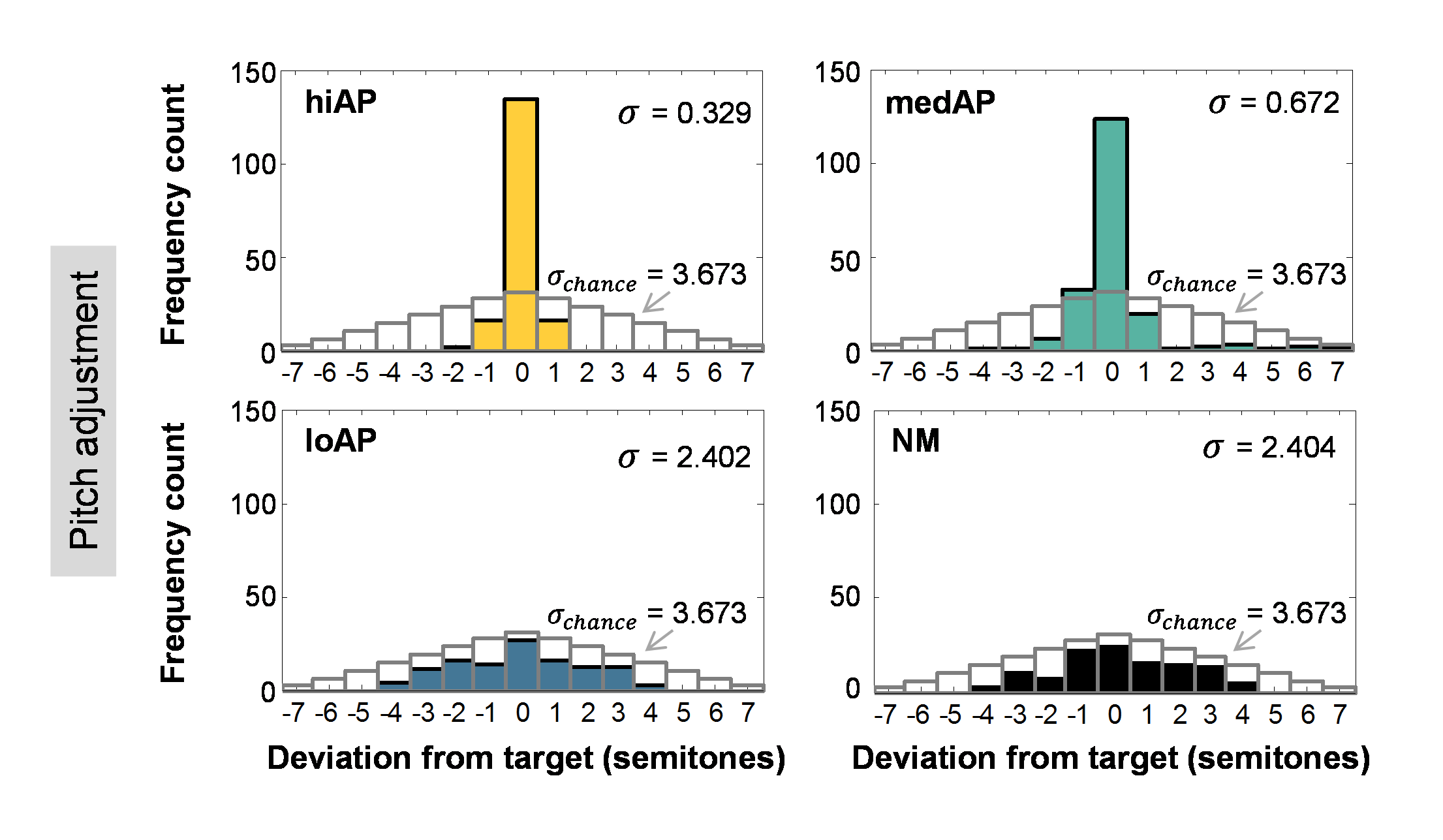


**Supplementary Figure 1**. Pitch adjustment distribution data for the high-AP, medium-AP, low-AP musicians, and non-musician groups. Gray distributions indicate chance performance simulated from a Monte-Carlo simulation.


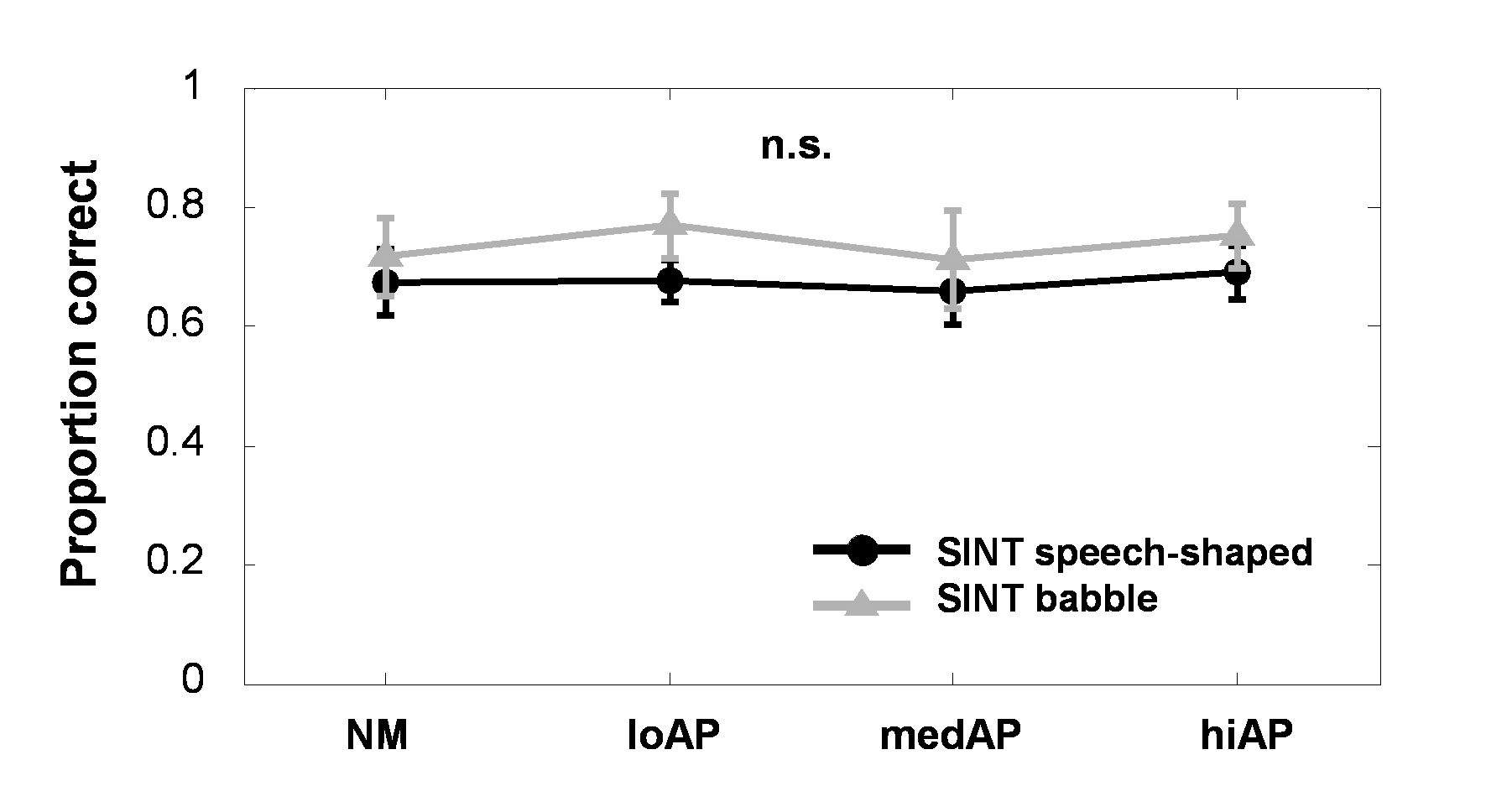


**Supplementary Figure 2**. Speech-in-noise performance under multi-babble noise (speech-shaped noise included for comparison) for different qAP-proficiency groups. There was no quasi-AP enhancement effect on SINT performance with multi-babble noise, *F*(3,50) = 2.102, *p* = 0.112.
